# Neuropharmacological Validation of *Clinopodium pulchellum* (Panizara): Unveiling the Anxiolytic and Antidepressant Mechanism via In Vivo Models and Molecular Docking

**DOI:** 10.3390/plants15101511

**Published:** 2026-05-15

**Authors:** Juan E. Valdiviezo-Campos, Ramiro Fiestas-Jacinto, Karyn A. Olascuaga-Castillo, Segundo G. Ruiz-Reyes, Susana R. Rubio-Guevara, Roger A. Rengifo-Penadillos, Junior F. Siguas-Peña

**Affiliations:** 1 Research in Natural Products, Programa Académico de Farmacia y Bioquímica, Facultad de Ciencias de la Salud, Universidad Privada Norbert Wiener, Lima 15046, Peru; juan.valdiviezo@uwiener.edu.pe (J.E.V.-C.); ramiro.fiestas@uwiener.edu.pe (R.F.-J.); 2 PHARMASCIENCE Research Group, Pharmacology Laboratory, School of Human Medicine, Universidad Privada Antenor Orrego, Trujillo 13001, Peru; kolascuagac1@upao.edu.pe (K.A.O.-C.); srubiog1@upao.edu.pe (S.R.R.-G.); 3 Department of Pharmacotechnics, Faculty of Pharmacy and Biochemistry, National University of Trujillo, Trujillo 13011, Peru; sruizr@unitru.edu.pe; 4 Department of Biochemistry, Faculty of Pharmacy and Biochemistry, National University of Trujillo, Trujillo 13011, Peru; rrengifo@unitru.edu.pe

**Keywords:** panizara, plus maze test, CUMS-forced swim test, neuropharmacological activity, essential oil, molecular docking

## Abstract

(1) Background: *Clinopodium pulchellum* (Kunth) Govaerts (Panizara) is an aromatic Andean medicinal plant traditionally used in Peru to manage nervous disorders, insomnia, and digestive complaints; however, its neuropharmacological properties remain poorly validated. This study aimed to evaluate the anxiolytic- and antidepressant-like effects of *C. pulchellum* and to characterize its phytochemical profile as supportive evidence. (2) Methods: The essential oil was obtained by hydrodistillation and analyzed using GC–MS and GC–FID. (3) Results: Fifteen volatile compounds were identified based on retention indices and mass spectral data, with β-caryophyllene (22.9%) and linalool (19.1%) as the most representative constituents, while other compounds were tentatively identified. The aqueous extract showed total phenolic and flavonoid contents of 34.15 mg GAE/g and 29.44 mg QE/g, respectively, and moderate antioxidant activity (DPPH = 2.36 mg TE/g; ABTS = 3.33 mg TE/g). In vivo assays revealed that EOCP at 200 mg·kg^−1^ significantly increased open-arm exploration in the elevated plus maze and reduced immobility time in the CUMS–forced swim test by 37% compared with the stress group, although the effect was lower than that of reference drugs. Molecular docking analysis indicated favorable binding affinities of β-caryophyllene, humulene, and aromandendrene with serotonergic and ion channel targets, while ADMET predictions suggested suitable pharmacokinetic properties. (4) Conclusions: These findings indicate that the observed neuropharmacological effects may be associated with the presence of bioactive terpenoids typical of Lamiaceae, supporting the traditional use of *C. pulchellum*. However, further studies are required to confirm the identity of uncommon constituents and to elucidate the mechanisms underlying its biological activity.

## 1. Introduction

The genus *Clinopodium* (family Lamiaceae) includes various aromatic species distributed throughout temperate and tropical regions of America, Europe, and Asia. These plants have been widely used in traditional medicine to treat central nervous system disorders such as anxiety, insomnia, and depression, as well as digestive and respiratory disorders [1]. In certain areas of Latin America, the use of *Clinopodium* correlates with cultural syndromes described as “fright,” “nerves,” or “shock,” terms that local populations interpret as expressions of emotional imbalance linked to anxiety and depressive disorders [2]. In addition to their medical relevance, these species have high biocultural value due to their culinary use and their role in sociocultural exchange in indigenous communities [3]. *Clinopodium pulchellum* (Kunth) Govaerts is an aromatic plant native to the Andes, recognized for its traditional use in indigenous communities in Peru and Ecuador. Although *Clinopodium pulchellum* has a well-established place in local ethnobotanical practice, published research on its chemical profile and pharmacological properties remains limited [4]. However, phytochemical research conducted on other species of the *Clinopodium* genus has reported a wide variety of secondary metabolites, such as flavonoids, terpenoids, essential oils, triterpenoids, alkaloids, and phenolic compounds [3,5,6,7]. These compounds are associated with antioxidant activity and with modulation of central nervous system function. Extracts of C. mexicanum demonstrated central nervous system depressant effects in animal models, indicating possible anxiolytic and antidepressant effects [4,8]. Analyses of the essential oil identified pulegone and menthone as principal constituents; both compounds display established antioxidant activity [9]. Monoterpenes and sesquiterpenes have been identified in the essential oils of *Clinopodium* gracile; these oils exhibit larvicidal and antioxidant activity, which highlights the therapeutic potential of the genus’s volatile compounds [10].

An analysis of *Clinopodium bolivianum* (muña) identified volatile components dominated by pulegone. The study evaluated the species’ potential for cosmetic applications, including shampoo, styling cream, and hair lotion, and reported product stability under accelerated conditions and notable antioxidant activity in the styling cream [11]. These results support the hypothesis that *C. pulchellum* could contain bioactive compounds with therapeutic potential. The chemical composition of essential oils from the *Clinopodium* genus can vary considerably between species depending on factors such as geographical location, stage of development, and environmental conditions [7]. However, monoterpenes such as pulegone, menthone, and limonene, as well as sesquiterpenes such as caryophyllene and germacrene D, are among the most common compounds [11]. These molecules are linked to a variety of biological activities, such as antimicrobial, antioxidant, and anti-inflammatory properties.

This study aims to provide neuropharmacological validation of *Clinopodium pulchellum* (Panizara) by investigating its anxiolytic and antidepressant-like effects through in vivo behavioral models and molecular docking analyses. Phytochemical and volatile profiling were used as supportive tools to identify bioactive constituents potentially responsible for these neuroactive effects. Validating the pharmacological properties of plants traditionally used in medicine facilitates the integration of ethnobotanical knowledge with modern experimental and computational approaches. This strategy not only provides scientific evidence supporting traditional uses but also contributes to the identification of plant-derived compounds with therapeutic potential, while promoting the recognition and preservation of Andean ethnobotanical heritage.

## 2. Results and Discussion

**Figure 1:**
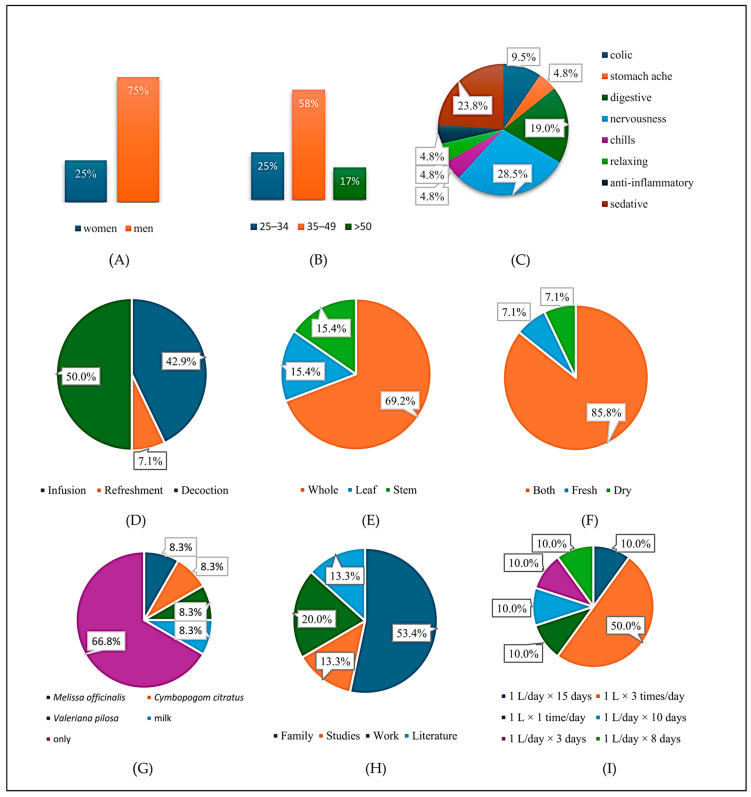
Ethnobotanical information for *Clinopodium pulchellum* collected from herbalists. Panels show: (**A**) gender; (**B**) age; (**C**) reported medicinal uses; (**D**) method of application; (**E**) plant part used; (**F**) conditions treated; (**G**) combinations with other species; (**H**) source of knowledge; (**I**) dosage.

The demographic analysis of the informants showed that 75% were women and 25% were men (Figure 1A), with a predominance of the 35–49 age group (58.3%) (Figure 1B). These results demonstrate the active participation of adult women in the conservation and transmission of ethnobotanical knowledge about *C. pulchellum*. The predominance of women in this field coincides with observations in rural communities, where women tend to be more knowledgeable about species close to their domestic environment, while men are more familiar with plants in forest areas [12]. This pattern reflects traditional gender roles, with women primarily responsible for family health. Similarly, the strong representation of working-age adults indicates that ethnobotanical knowledge is not confined to older generations but persists among economically active cohorts [13].

These findings underscore the importance of considering gender and age in ethnobotanical studies, as well as the need to strengthen the role of women and adults in the transmission of traditional knowledge. In terms of medicinal uses, *C. pulchellum* is mainly used to treat nervousness (28.5%), as a sedative (23.8%), and for digestive disorders (19%) (Figure 1C). Other less frequent uses include relief from colic (9.5%), stomach pain, chills, and relaxing and anti-inflammatory effects (4.8% each). This confirms its therapeutic versatility, acting on conditions of the nervous and digestive systems. The high percentage of use as a sedative supports its traditional application in cultural syndromes such as “fright” or “shock” in high Andean communities [2,8], in addition to its relevance in the management of emotional states.

Regarding preparation methods, decoction was the most common (50%), followed by infusion (42.9%) and soft drink (7.1%) (Figure 1D). This pattern is consistent with ethnobotanical practices documented in other regions, such as the province of Kerman (Iran), where oral administration by decoction predominates [14]. Similarly, in species of the same genus, 65% of informants report decoction as the main method [15].

According to Figure 1E, the most frequently used plant parts were leaves and stems (15.4% each), followed by the whole plant (69.2%). Comprehensive use reflects a holistic approach to traditional medicine, which seeks to take advantage of the synergy between different plant organs. This pattern coincides with the use of *C. nepeta* in digestive disorders [16], while *C. chinense* is preferably used in leaves and flowers due to its higher concentration of bioactive metabolites [17].

The observed variation within the genus underscores the influence of cultural context and the need for comprehensive evaluation of traditional uses in phytochemical investigations. Regarding plant material, informants reported the use of fresh (7.1%) and dried (7.1%) material, followed predominantly by the combined use of both forms (85.8%) (Figure 1F). This practice demonstrates empirical knowledge of preservation and efficacy, ensuring year-round availability. Recent studies have shown that drying can increase phenolic content and antioxidant activity [18]. Similarly, related species such as *C. tomentosum* and *C. bolivianum* are used in both fresh and dried forms to treat respiratory and digestive ailments and for antibacterial purposes [19]. 66.8% of respondents reported using *C. pulchellum* exclusively, while 33.2% combined it with species such as *C. citratus*, *M. officinalis*, and *V. pilosa* (Figure 1G). This individualized use reflects confidence in its therapeutic efficacy, while combinations seek to enhance its anxiolytic and digestive effects through synergy, a common practice in traditional medicine [20,21,22,23].

Sources of knowledge indicate that 53.4% of informants learned about the plant through family transmission, followed by empirical learning (20%), formal education (13.3%), and written sources (13.3%) (Figure 1H). This pattern reaffirms the predominance of oral knowledge while also indicating increasing integration with formal education and scientific documentation [24,25]. Finally, dosage varies according to the informant’s experience, the ailment, and the type of preparation. The most common regimen is to ingest 1 L daily for 3 days (50%), followed by more than 10 days (20%) and 15 days (10%) (Figure 1I). This variability reflects the empirical nature of traditional medicine, influenced by metabolite concentration, environmental conditions, and post-harvest handling [26,27].

**Figure 2:**
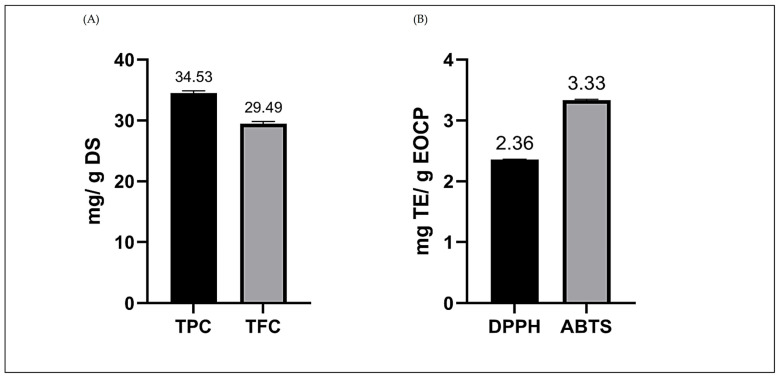
(**A**) Total phenol and flavonoid content in non-volatile extract and (**B**) antioxidant capacity of essential oil from the species *C. pulchellum*. DS: dry sample, EOCP: *C. pulchellum* essential oil. TE: Trolox equivalent.

The non-volatile extract of *Clinopodium pulchellum* contained 34.15 ± 0.16 mg GAE/g of total phenolic compounds and 29.44 ± 0.69 mg QE/g of total flavonoids (Figure 2A). The higher concentration of phenols suggests stable extraction, while flavonoids, due to their thermo- and photosensitive nature, may be affected by variables such as cooking time and the plant/water ratio used.

The values obtained (TPC = 34.15 mg GAE/g; TFC = 29.44 mg QE/g) fall within an intermediate range within the genus *Clinopodium* and the family Lamiaceae. Although there are no specific previous reports on *C. pulchellum*, studies on related species allow useful comparisons to be made. In *C. vulgare*, Bektašević et al. [28] identified concentrations of rosmarinic acid (26.63–34.21 mg/g) and ellagic acid (23.11–29.31 mg/g), values like those observed in this study, suggesting a comparable richness in individual phenolic acids. In contrast, Tubón et al. [29] reported much higher contents in *C. tomentosum* (TPC = 140.15 ± 0.12 mg GAE/g; TFC = 97.38 ± 0.62 mg QE/g) in ethanolic extracts, reflecting the influence of both the type of solvent (ethanol, which is more efficient for polar compounds) and the species.

Similarly, Guzzo et al. [30] found TPC values between 5.97 ± 0.33 and 51.47 ± 0.62 mg GAE/g, and TFC values between 11.21 ± 0.53 and 43.86 ± 0.63 mg RE/g in *C. nepeta* subsp. *glandulosum,* depending on the solvent used. The aqueous and ethanol/water extracts showed levels similar to those obtained here for *C. pulchellum*, reinforcing its position as a species moderately rich in phenolic metabolites. Similarly, Jaramillo et al. [31] reported higher values in *C. nubigenum*, with TPC ranging from 95.0 ± 3.7 to 184.1 ± 17.8 mg GAE/g, differences attributable to the use of modern extraction technologies, such as supercritical fluids or ultrasound, which increase the yield of secondary metabolites. In *C. nepeta*, Çelik et al. [32] recorded a TPC of 61 mg GAE/g and a remarkable antioxidant capacity (IC_50_ DPPH = 1.82 mg/mL), exceeding the values of *C. pulchellum* and confirming the influence of the plant matrix and extraction method on bioactivity.

This variability in flavonoid content reflects their sensitivity to heat, pH, and solvent polarity. Gentle extraction methods such as infusion better preserve these metabolites, whereas decoction, which involves prolonged exposure to high temperatures, can promote their degradation. Studies on Fraxinus excelsior have shown that infusions retain higher concentrations of phenolic compounds than decoctions, consistent with the thermal lability of antioxidant phytochemicals. Similarly, the choice of solvent is decisive: solvents of intermediate polarity favor the extraction of flavonoids [31,33,34]. Overall, *C. pulchellum* shows a stable phytochemical profile consistent with its traditional use, which could be optimized by adjusting the extraction conditions.

In relation to antioxidant activity (Figure 2B), the essential oil of *C. pulchellum* presented 2.36 mg TE/g (DPPH) and 3.33 mg TE/g (ABTS). The higher capacity observed in ABTS compared to DPPH is consistent with reports of other Lamiaceae oils, where the ABTS assay shows greater sensitivity to hydrophilic compounds. In *C. brownei*, Noriega et al. [35] observed an IC_50_ ABTS = 0.06 mg/mL and IC_50_ DPPH = 1.77 mg/mL, confirming this trend (ABTS > DPPH). Similarly, Tapia et al. [36] reported an IC_50_ (DPPH) = 2288 μg/mL and IC_50_ (ABTS) = 30.87 μg/mL for *C. pulchellum*, results consistent with the pattern obtained in the present study.

When compared to *Thymus* species, whose essential oils contain between 11.69 and 28.23 mg TE/g (ABTS) and IC_50_ DPPH = 0.60–9.23 mg/mL, the values for *C. pulchellum* are at a low-intermediate level [37]. This suggests moderate antioxidant capacity, attributable to its chemical composition dominated by monoterpenes and phenolic derivatives in intermediate concentrations.

The organoleptic and physicochemical parameters (Table 1) of EOCP are consistent with those typical of Lamiaceae oils [7,35,38], which are clear, pale yellow liquids with herbal aromas. The relative density and refractive index indicate good purity. The slightly acidic pH supports its stability. The results suggest that this oil is of adequate quality and has characteristics consistent with other similar aromatic species.

Chromatographic analysis of EOCP identified fifteen compounds (Table 2), dominated by 3-methyl-2-(2-methyl-2-butenyl) furan (rosefuran, 42.04%), β-caryophyllene (22.92%), and linalool (19.13%), which together accounted for 84.09% of the total. This composition reveals a mixed profile characterized by the predominance of sesquiterpenes (31%), followed by oxygenated monoterpenes (19.5%) and hydrocarbon monoterpenes (7.4%), a distribution similar to that described in other species of the genus *Clinopodium* and the family Lamiaceae, where terpenoids such as pulegone, menthone, linalool, β-caryophyllene, humulene, and germacrene D are commonly reported as major constituents [3,32].

However, the compound tentatively identified as 3-methyl-2-(2-methyl-2-butenyl) furan (rosefuran) is not typically reported as a major constituent in *Clinopodium* species or in Lamiaceae essential oils. From a chemotaxonomic perspective, this finding should therefore be interpreted with caution and may reflect a particular chemotype of *C. pulchellum* influenced by ecological, geographical, or phenological factors rather than a characteristic marker of the genus.

Lamiaceae species are typically characterized by essential oils rich in monoterpenes and sesquiterpenes such as pulegone, menthone, linalool, and β-caryophyllene, which are consistently reported in previous studies across different *Clinopodium* species [1,6,7]. In contrast, the occurrence of furanoid compounds as major constituents is uncommon and should therefore be interpreted cautiously from a chemotaxonomic perspective.

This type of compound has been associated with antioxidant and neuroprotective activity in other Lamiaceae [28]. β-Caryophyllene, reported in *C. nubigenum* and *C. candidissimum*, is notable for its anti-inflammatory, analgesic, and endocannabinoid system-modulating effects, and its high concentration in *C. pulchellum* positions it as a differential chemical marker, exceeding the usual levels (1.9–5.1%) described in other species [31,32]. The sesquiterpene (E)-caryophyllene derivatives, compounds described in *C. brownei*, are responsible for antioxidant activity [35].

Likewise, linalool reached a high proportion (19.13%), comparable to that recorded in *C. nepeta* and *C. tomentosum* (up to 58.43%), which supports its link to the traditional use of the species as a relaxing and sedative plant [29,38]. Minor sesquiterpenes, such as humulene and germacrene D, were also detected at levels like those reported for *C. macrostemum* and *C. candidissimum* (up to 19.4%) [39], confirming the interspecific phytochemical similarity within the genus.

For their part, hydrocarbon monoterpenes, including β-pinene and limonene, were associated with antioxidant and antimicrobial activities and were within the ranges observed in other *Clinopodium* species (0.3–5.5% and 0.9–3.7%, respectively) [3,7,39]. Finally, 1,8-cineole (eucalyptol) was identified in a low proportion (0.43%), lower than that reported in *C. nepeta* and *C. tomentosum* (7.94–22.8%) [7,38], suggesting biochemical differences specific to *C. pulchellum*.

Table 3 shows the anxiolytic activity as a function of the time spent and number of entries into the open and closed arms in the elevated plus maze (EPM) Test. This model evaluates rats’ natural aversion to elevated and open spaces [40], as well as the exploratory behavior present in this species in novel environments [41]. The length of stay and number of entries into the open arms are directly related to reduced anxiety levels in the experimental animals, associated with neophobia, exploration, and approach/avoidance conflict, making EPM a model of spontaneous, unconditioned behavioral conflict [40]. It is observed that the longest time spent and the highest number of entries into the open arms correspond to the reference drug (diazepam, 1 mg/kg BW) with 207.2 ± 12.57 s spent (*p* < 0.05) and 12.8 ± 0.79 entries (*p* < 0.05), respectively. Diazepam is the reference anxiolytic drug in EPM, producing an increase in exploration of the open arms [40].

In contrast, the group that received only PTZ (20 mg/kg BW) showed the lowest dwell time and number of entries of all groups (44.1 ± 5.73 s and 2.9 ± 0.23 entries, respectively), which is consistent with the findings of Squires et al. [42], who reported that the administration of subconvulsant doses (<25 mg/kg BW) causes anxiogenic behavior in rats by blocking the postsynaptic actions of the inhibitory neurotransmitter gamma-aminobutyric acid (GABA). Among the essential oils, the highest values for both parameters were in the EOCP group (200 mg/kg) with a residence time and number of entries into the open arms of 170.7 ± 4.93 s (*p* < 0.05) and 9.1 ± 0.50 times (*p* < 0.05), respectively.

Although the administration of EOCP resulted in more frequent entries and longer stays in the open arms, the resulting behavioral profile did not strictly align with the traditional sedative-anxiolytic response seen with diazepam. The open-to-closed arm ratio points away from simple central nervous system depression [43]. A more plausible interpretation is a dual mechanism where anxiolytic-like effects occur alongside increased exploratory behavior or hyperlocomotion, a complex dynamic frequently encountered with multi-component terpenoid blends [44,45].

The chronic unpredictable mild stress (CUMS) model significantly increased immobility time in the forced swim test (FST), confirming the successful induction of a depressive-like phenotype. Administration of EOCP reduced immobility time, with a statistically significant effect observed only at the highest tested dose (200 mg/kg; 128.7 ± 4.41 s) compared with the CUMS group (204.3 ± 8.26 s), although the magnitude of the effect remained lower than that produced by fluoxetine (86.5 ± 2.91 s) (Table 4). These findings indicate that EOCP exhibited a measurable antidepressant-like effect at the highest tested dose, suggesting the presence of a pharmacological threshold required to elicit behavioral improvement. Similar behavioral modulation has been reported for essential oils of other species within the Lamiaceae family with neuroactive properties [46,47].

Previous studies on species of the same genus support these results. *Clinopodium mexicanum* has demonstrated antidepressant and anxiolytic effects in animal models, attributed to modulation of the GABAergic and serotonergic systems [4,48]. In EOCP, the presence of β-caryophyllene (22.9%) and linalool (19.1%) contributed to the observed effect. β-caryophyllene acts as a selective CB_2_ receptor agonist, reducing immobility in the FST and restoring the Brain-Derived Neurotrophic Factor (BDNF), a key neurotrophin involved in neuronal survival and synaptic plasticity that is typically downregulated in chronic stress models [49,50]. Linalool, on the other hand, exerts antidepressant effects mediated by the serotonergic pathway (5-HT_1_A) and increased dopamine and serotonin in the hippocampus [51,52].

The magnitude of the EOCP effect (37% reduction compared to the CUMS group) is comparable to that of essential oils from *Thymus vulgaris* and *Lavandula angustifolia*, whose main monoterpenes also act on monoaminergic and endocannabinoid receptors [46,49]. Taken together, these findings suggest that the antidepressant-like effect of *C. pulchellum* may be associated with the presence of neuroactive terpenoids, particularly β-caryophyllene and linalool, which have been reported in previous studies to modulate neurotransmission and reduce the behavioral effects of chronic stress.

While the present findings provide a valuable initial neuropharmacological validation for *Clinopodium pulchellum*, further rigorous experimental validation is required. Future in vivo studies should focus on determining the precise effective dose (ED_50_), ensuring the rigorous standardization of the essential oil, and executing comprehensive safety and toxicological evaluations. Furthermore, evaluating the behavioral effects of the primary isolated compounds proposed in this study will be essential to accurately map their specific mechanisms of action.

In silico analysis using the SwissADME and pkCSM platforms allowed us to evaluate the physicochemical and pharmacokinetic properties of the major compounds in EOCP.

**Figure 3:**
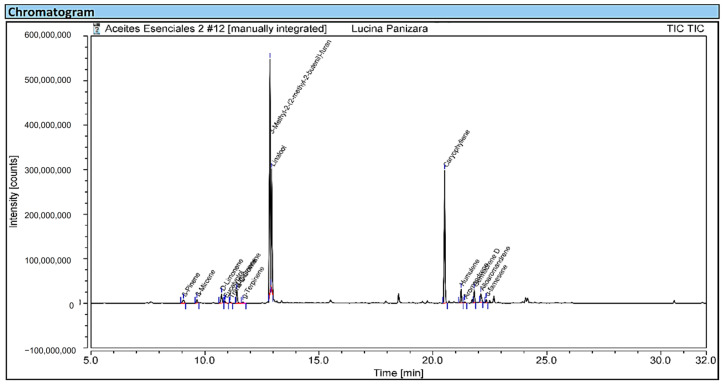
GC-MS total ion chromatogram (TIC) of the essential oil of *Clinopodium pulchellum* (EOCP).

A total of 15 volatile compounds identified by GC–MS analysis (Figure 3), including β-caryophyllene, linalool, humulene, germacrene D, and 3-methyl-2-(2-methyl-2-butenyl) furan, were selected for in silico evaluation. All metabolites met the criteria of Lipinski’s Rule of Five, a key parameter in rational drug design and the prediction of oral bioavailability. The results indicated molecular weights (MW) ≤ 500 g/mol, molar refractivity (MR) indices between 40 and 130, log P (Octanol/Water) values < 5, hydrogen bond donors (HBD) ≤ 5, and hydrogen bond acceptors (HBA) ≤ 10, as summarized in Table 5. This profile suggests that the volatile compounds of EOCP possess characteristics typical of molecules with pharmacological potential, capable of crossing biological membranes and exhibiting adequate intestinal absorption.

These results are consistent with previous studies by Benkhaira et al. [7], who demonstrated that the compounds evaluated comply with Lipinski’s Rule, showing good permeability, solubility, and structural similarity with reference ligands. Similarly, Lipinski et al. [53] highlighted that compounds that satisfy these parameters tend to have greater oral bioavailability and metabolic stability, which supports the favorable pharmacokinetic potential of the components of EOCP.

Analysis of ADME and toxicity profiles confirmed that all 15 molecules showed high human intestinal absorption (HIA) of over 94%. In addition, they exhibited favorable blood–brain barrier (BBB) permeability with log BB values between 0.3 and 0.9 and central nervous system (CNS) diffusion properties between −1 and −3 log PS. Regarding interactions with the cytochrome P450 enzyme system, no molecules were predicted to have inhibitory effects, except for compounds C1, C2, C12, C13, C14, and C15, which could inhibit the CYP1A2 isoenzyme. On the other hand, only compound C3 was identified as a potential cytochrome substrate. Regarding toxicity, all molecules were classified as non-mutagenic according to the AMES test, and no hepatotoxicity was predicted. However, a possible skin sensitization effect was identified for compounds C1, C2, C8, C9, C10, C11, C12, and C13, as detailed in Table 6.

**Figure 4:**
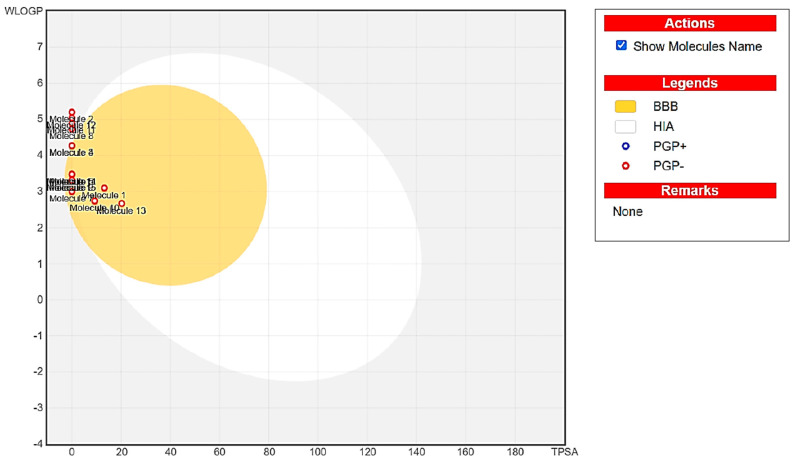
The BOILED-Egg model of fifteen chemical compounds of EOCP.

The BOILED-Egg predictive model (Figure 4), based on topological surface area (TPSA) and lipophilicity (WLOGP) parameters, supports these findings for assessing absorption and distribution, indicating that all chemical molecules analyzed are located within Egan’s yellow oval, with compounds C3 and C4 at the margins of this zone, while C2, C8, C11, and C12 are outside the white and yellow regions, indicating a low probability of effective absorption and distribution. This suggests that most compounds have a high potential to passively cross the blood–brain barrier, positioning them as promising candidates for applications in the central nervous system (CNS).

The molecular docking study allowed the prediction of interactions between fifteen volatile compounds from EOCP and three target proteins related to antioxidant (2CDU), anxiolytic (4UUJ), and antidepressant (5I6X) activity. The results showed binding affinities comparable to or higher than those of the reference ligands: ascorbic acid (−6.4 kcal/mol), diazepam (−6.0 kcal/mol), and imipramine (−8.7 kcal/mol). The control ligands ascorbic acid, diazepam, and imipramine showed molecular interactions consistent with their receptors. Diazepam established hydrogen bonds with Gly42 and hydrophobic contacts with Pro41, Val93, and Pro154; imipramine exhibited hydrophobic interactions with Tyr95, Ile172, and Phe341, as well as π interactions with Tyr176; and ascorbic acid formed hydrogen bonds with Thr9, His10, and Ala11. These results suggest favorable molecular affinity and stable binding conformations, supporting the potential bioactivity of these compounds, consistent with previous studies [54,55].

**Figure 5:**
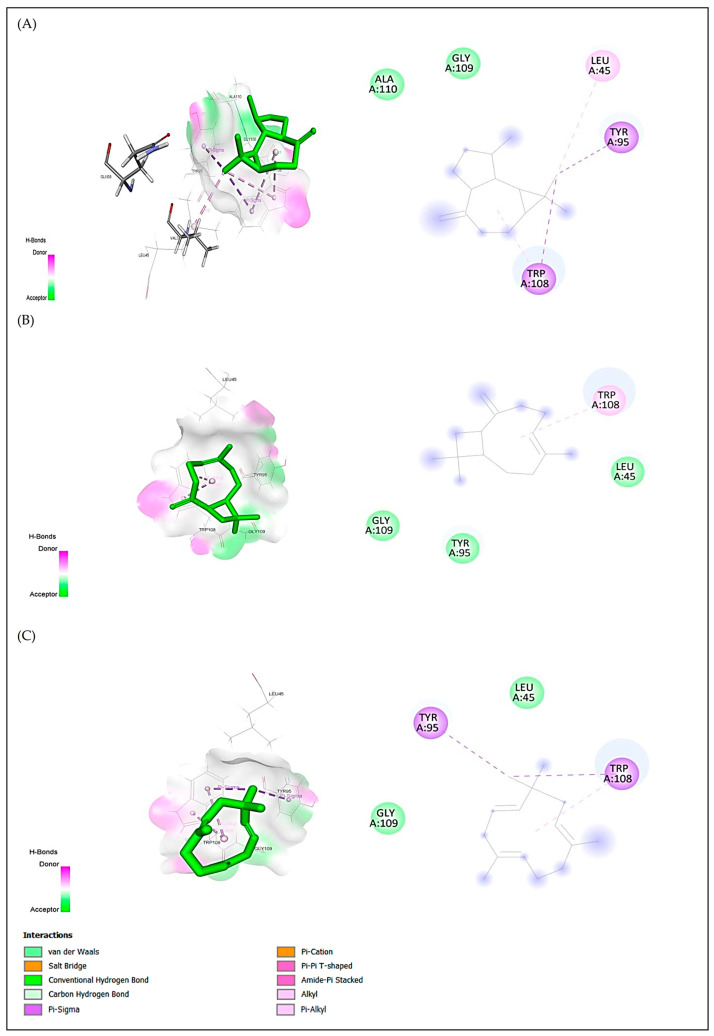
2D and 3D visualizations of intermolecular interactions for three major compounds ((**A**) aromandendrene, (**B**) caryophyllene and (**C**) humulene) in complex with the potassium channel protein (4UUJ. pdb) from the bacterium *Streptomyces lividans*. The ligand is located within the active site of the protein, where it interacts with key amino acid residues, including Tyr95, Trp108, and Leu45. Binding is primarily mediated by π-alkyl interactions (pink dashed lines) and π-sigma interactions (purple dashed lines), contributing to hydrophobic stabilization of the ligand–protein complex. Additional residues such as Gly109 and Ala110 are involved in van der Waals interactions (light green regions), facilitating ligand accommodation within the binding pocket. Hydrogen bond donor and acceptor regions are indicated by magenta and green surfaces, respectively, highlighting the contribution of polar interactions to complex stability.

**Figure 6:**
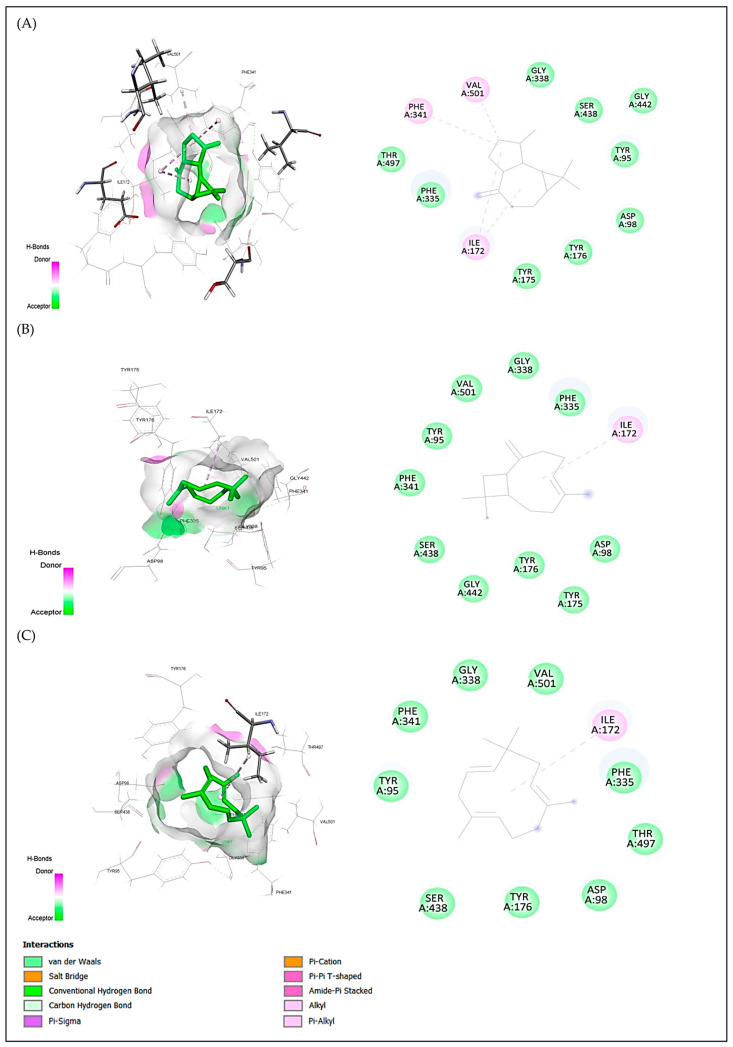
2D and 3D visualizations of intermolecular interactions for three major compounds ((**A**) aromandendrene, (**B**) caryophyellene and (**C**) humulene) in complex with the serotonin transporter protein (5I6X.pdb). The ligand is positioned within the binding pocket and interacts with surrounding amino acid residues through hydrophobic contacts. Key interactions are observed with residues such as Ile172, Val501, and Phe341 via alkyl interactions (pink dashed lines), contributing to the stabilization of the ligand–protein complex. Additional residues including Tyr95, Phe335, Gly338, Ser438, and Asp98 are involved in van der Waals interactions (green regions), facilitating ligand accommodation within the binding site. These interactions indicate that hydrophobic forces play a dominant role in ligand binding.

**Figure 7:**
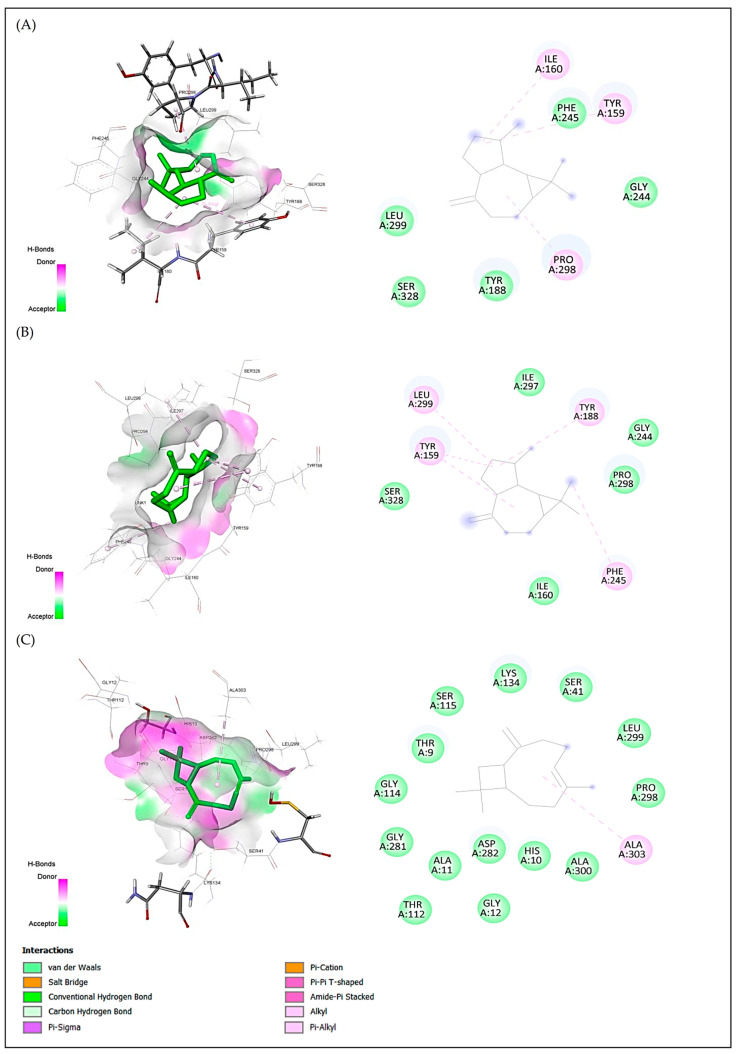
2D and 3D visualizations of intermolecular interactions for three major compounds ((**A**) alloaromandendrene, (**B**) aromandendrene and (**C**) caryophyllene) in complex with NADPH oxidase protein (2CDU.pdb) from *Lactobacillus Sanfranciscensis*. The ligand is positioned within the binding pocket and interacts with surrounding amino acid residues primarily through hydrophobic contacts. Key interactions are observed with residues such as Ile160, Tyr159, Pro298, and Phe245 via alkyl interactions (pink dashed lines), contributing to stabilization of the ligand–protein complex. Additional residues, including Leu299, Ser328, Gly244, Tyr188, and Ala303, are involved in van der Waals interactions (green regions), facilitating ligand accommodation within the binding site. The predominance of nonpolar interactions and the observed interaction distances suggest a stable binding conformation driven mainly by hydrophobic forces.

For anxiolytic activity, the best docking scores were obtained for aromandendrene, caryophyllene, and humulene with binding energies of −5.5, −5.3 and −5.2 kcal/mol, respectively. For antidepressant activity, the best docking scores were obtained for humulene, aromandendrene and caryophyllene with binding energies of caryophyllene of −8.1, −7.9 and −7.8 kcal/mol, respectively. For antioxidant activity, the best docking scores were obtained for alloaromandendrene, aromandendrene, and caryophyllene with binding energies of −6.8, −6.8 and −6.7 kcal/mol, respectively (Table 7). These compounds met all the criteria for similarity to drugs; aromandendrene showed pi-sigma interactions with Tyr98 and Trp108 and hydrophobic interaction with Leu45 for protein 4UUJ (Figure 5); humulene showed hydrophobic interaction with Ile172 for protein 5I6X (Figure 6); and aromandendrene showed hydrophobic interactions with Tyr159(2), Tyr188, Phe245, and Leu299 for protein 2CDU (Figure 7).

The affinity observed for humulene (−8.1 kcal/mol) and β-caryophyllene (−7.8 kcal/mol) for the 5I6X receptor is consistent with studies linking this type of sesquiterpene to modulation of the CB_2_ endocannabinoid system and the monoaminergic pathway, contributing to the antidepressant effect observed in vivo [56]. Fonseca et al. [46] also reported, through docking, that oxygenated monoterpenes and sesquiterpenes from essential oils have a high affinity for serotonergic receptors (5-HT_1_A and SERT), with binding energies between −6.5 and −8.3 kcal/mol, ranges consistent with those obtained for *C. pulchellum* compounds.

Similarly, aromandendrene (−5.5 kcal/mol) showed significant affinity for the potassium channel (4UUJ), interacting with residues Tyr98, Trp108, and Leu45. This type of binding is associated with the modulation of neuronal conductance and reduction in excitability, mechanisms involved in the anxiolytic effect of Lamiaceae essential oils [57]. In the antioxidant context, the negative energies of the complexes formed by aromandendrene and β-caryophyllene (−6.8 and −6.7 kcal/mol) with the 2CDU protein support their ability to stabilize free radicals, a result consistent with the coupling of 1,8-cineole reported by Benkhaira et al. [7] in *Clinopodium nepeta*.

The pattern of interactions found coincides with the review by Jarończyk et al. [58], which highlights that several natural compounds with antidepressant potential act through multiple affinities toward serotonergic targets and ion channels, a behavior characteristic of multitarget ligands. In this sense, the energy values and similarity in the types of bonds predicted for *C. pulchellum* compounds reinforce their role as multitarget candidates capable of modulating oxidative and neurochemical pathways associated with depression.

There are no previous studies describing the molecular coupling of volatile compounds from *Clinopodium pulchellum* with proteins related to antioxidant, anxiolytic, and antidepressant activity. This lack of information underscores the novelty of this research, which provides computational evidence of such interactions. The results of the docking study support the experimental findings and expand knowledge about the therapeutic potential of *C. pulchellum*.

## 3. Materials and Methods

### 3.1. Chemicals

Ethanol (Spectrum Chemical, New Brunswick, NJ, USA), distilled water (Dropaksa, Lima, Peru), 2,2-diphenyl-1-picrylhydrazyl (DPPH), 2,2-azinobis-3-ethylbenzothiazoline-6-sulfonic acid (ABTS) and potassium persulfate (Sigma-Aldrich, St. Louis, MO, USA) were used. Trolox (6-hydroxy-2,5,7,8-tetramethylchroman-2-carboxylic acid), gallic acid and quercetin (Sigma-Aldrich, St. Louis, MO, USA) were used as standards.

### 3.2. Ethnobotanical Study

A descriptive ethnobotanical study was conducted in the wholesale markets of Trujillo, La Libertad region, including a total of 64 medicinal plant traders selected by random sampling. Trained interviewers administered semi-structured questionnaires to participants after obtaining informed consent. Collected data included vernacular plant names, reported medicinal uses, methods of application, plant parts used, conditions treated, combinations with other plants, dosage, and sources of knowledge. This methodology followed the approach proposed by Valdiviezo-Campos et al. [59]. The study received approval from the Ethics Committee of the Norbert Wiener Private University (registration code no.: A0055-2025; approval date: 12 September 2025).

### 3.3. Plant Material

The species studied was *Clinopodium pulchellum* (Kunth) Govaerts. The plant material was collected on 16 August 2025, during the full flowering stage (anthesis). This timing coincides with the peak of the dry season in the Huamachuco District, Sánchez Carrión Province, La Libertad Region (7°50′07″ S, 78°01′59″ W; 3458 m a.s.l.). This phenological stage and the specific seasonal conditions (high solar radiation and water stress typical of the Andean winter) were selected to ensure the maximum accumulation of secondary metabolites. A complete specimen was pressed and prepared according to the standard protocols of the *Herbarium Truxillense* (HUT) and was taxonomically identified under registration No. 60830.

### 3.4. Essential Oil Extraction

The essential oil of *Clinopodium pulchellum* (EOCP) was obtained by hydrodistillation in triplicate using a 6 L Clevenger-type apparatus. A total of 6 kg of fresh leaves, collected on 16 August 2025 (flowering stage), were air-dried to obtain 1.82 kg of moisture-free biomass. The extraction was carried out in three batches of 606.7 g of dried plant material each, using a plant-to-water ratio of 1:8 (*w*/*v*) for 3 h. The oil volumes recovered in each run (1.6, 1.9, and 2.0 mL) yielded an average of 0.302 ± 0.034% (*v*/*w*), calculated on a dry weight basis. The EOCP was dried over anhydrous Na_2_SO_4_ and stored at 4 °C until further analysis. The aqueous fraction containing nonvolatile components was retained in amber containers at 4 °C for subsequent analysis.

### 3.5. Sensory Characteristics and Physicochemical Properties

After several hydrodistillations to obtain a sufficient amount of essential oil, the sensory characteristics and physicochemical properties were determined. The sensory characteristics and physicochemical properties of EOCP (appearance, color, odor, taste, density, refractive index, and acidity) were analyzed. All parameters were determined according to the International Pharmacopoeia method [60]. Relative density was calculated with a 5 mL pycnometer at 20 °C. The refractive index was determined with a digital ABBE refractometer (accuracy ±0.002). pH was measured with a pH meter (WTW^®^ model 7110, Weilheim, Germany). The total volume of 5.5 mL of EOCP corresponded to the pooled oil obtained from three independent hydrodistillation batches. Specifically, the volumes recovered per batch (606.7 g of dry biomass) were 1.6, 1.9, and 2.0 mL, resulting in a mean yield of 0.302% ± 0.034% (*v*/*w*). For pH determination, an aliquot of the essential oil was transferred to a beaker, and the electrode was immersed directly in the sample to record the pH values.

### 3.6. Identification of Volatile Components

The EOCP was analyzed using a Shimadzu 2010 GC-FID and GC-MS system (Shimadzu Corporation, Kyoto, Japan).

Both GC–FID and GC–MS analyses were performed under identical chromatographic conditions to ensure consistency in compound identification and quantification. A capillary column (HP-5MS, 5% phenyl-95% dimethylpolysiloxane; 30 m × 0.25 mm i.d., film thickness 0.25 μm) was employed. Nitrogen served as the carrier gas at a flow rate of 46.5 mL/min. Oven, injector, and detector temperatures were set to 280 °C, 280 °C, and 290 °C, respectively. For GC-FID analysis, split injection was used with 0.2 μL of pure oil and a split ratio of 10; GC-MS analyses were performed in split mode with a split ratio of 100. The injector, ion source, and GC-MS interface temperatures were 280 °C, 230 °C, and 270 °C, respectively. Mass spectra were acquired at 70 eV over the mass range 35–650 u. Component identification was based on a combination of retention indices (RI) calculated from a homologous series of n-alkanes (C9–C31), comparison of mass spectra with NIST (version 4.1) and Wiley (7th edition) libraries, and comparison with retention index values reported in the literature [61]. Additionally, fragmentation patterns were analyzed and compared with previously reported data to improve the reliability of compound identification.

### 3.7. Total Phenol and Flavonoid Content

Total phenol and flavonoid contents of the nonvolatile extract were quantified according to Valdiviezo-Campos et al. [62], with minor modifications.

Total phenolic content (TPC) was measured using the Folin–Ciocalteu method. Briefly, 100 µL of extract was mixed with diluted Folin–Ciocalteu reagent (1:10) and sodium bicarbonate (7.5% *w*/*v*), adjusted to 10 mL, and incubated for 60 min in the dark. Absorbance was read at 765 nm, and results were expressed as mg gallic acid equivalents per gram of dry plant material (mg GAE/g DS) using a gallic acid calibration curve. Total flavonoid content (TFC) was determined by the aluminum chloride method. 200 µL of extract was mixed with aluminum chloride (10% *w*/*v*) and sodium acetate (1 M), adjusted to 10 mL, and incubated for 30 min in the dark. Absorbance was measured at 430 nm, and results were expressed as mg quercetin equivalents per gram of dry plant material (mg QE/g DS) using a quercetin calibration curve. All measurements were performed in triplicate.

### 3.8. Antioxidant Activity Evaluation

#### 3.8.1. DPPH Radical Scavenging Activity

The free-radical scavenging activity was determined using the DPPH (2,2-diphenyl-1-picrylhydrazyl) assay with minor modifications. A 0.1 mM DPPH stock solution was prepared in 96% ethanol. In 10 mL volumetric flasks, 100 µL of the oil, diluted in 0.3% DMSO, was mixed with the DPPH solution, gently shaken, and incubated in the dark for 30 min at room temperature. Trolox was used as the calibration standard at concentrations of 3–30 µM. Absorbance was measured at 517 nm using a Peak Instruments C7000V spectrophotometer (Peak Instruments, Houston, TX, USA), with each determination performed in triplicate. Results are expressed as mg of Trolox equivalents per 100 g of dry plant material (mg TE/100 g), calculated from the antioxidant activity measured in the essential oil and normalized according to the essential oil yield relative to the dry plant biomass [63].

#### 3.8.2. ABTS Radical Scavenging Activity

The free-radical scavenging activity was determined using the ABTS (2,2′-azino-bis-3-ethylbenzothiazoline-6-sulfonic acid) assay with minor modifications. The stock solution was prepared by mixing equal volumes of ABTS (7 mM) and potassium persulfate (K_2_S_2_O_8_, 2.45 mM), incubating the mixture in the dark for 16 h, and then diluting with 50% ethanol to obtain the working solution. In 10 mL volumetric flasks, 100 µL of the oil, diluted in 0.3% DMSO, was mixed with the ABTS solution, gently shaken, and incubated in the dark for 30 min. Trolox was used as the calibration standard at concentrations of 3–20 µM. Absorbance was measured at 734 nm using a Peak Instruments C7000V spectrophotometer, with each determination performed in triplicate. Results are expressed as mg of Trolox equivalents per 100 g of dry plant material (mg TE/100 g), calculated from the antioxidant activity measured in the essential oil and normalized according to the essential oil yield relative to the dry plant biomass [63].

### 3.9. Anxiolytic Activity Assessment

#### Elevated Plus Maze (EPM)

The elevated plus maze (EPM) is a validated model for assessing anxiety-related behavior in rodents. It consists of four arms: two open (30 × 6 cm) and two closed (35 × 6 × 15 cm), constructed from black Plexiglas, connected by a central open platform (5 × 5 cm), and elevated 55 cm above the floor [64]. The open arms were bordered by raised edges (3 mm high, 1 mm thick) [65]. To evaluate the involvement of the GABAergic system, sixty mice were randomly assigned to six groups (*n* = 10) and pretreated intraperitoneally with vehicle (negative control), pentylenetetrazole 20 mg/kg (positive control), diazepam 1 mg/kg (reference drug), or EOCP at 50, 100, or 200 mg/kg [42,43]. Twenty minutes post-treatment, each mouse was placed at the center of the maze, facing an open arm, and allowed to explore freely for 5 min [44]. An arm entry was defined as the placement of all four paws within the arm. Sessions were recorded using a digital overhead camera (Logitech Europe S.A., Lausanne, Switzerland). The number of entries and time spent in open and closed arms were quantified. Tests were conducted in a quiet room under dim lighting conditions [45]. After each session, the apparatus was cleaned with a paper towel moistened with a mixture of ethanol, detergent, and water. Anxiety-related parameters were calculated as the percentage of open arms entries (OE% = OE/(OE + CE) × 100%) and the percentage of time spent in open arms (OT% = OT/(OT + CT) × 100%) [66].

### 3.10. Assessment of Antidepressant Activity

#### 3.10.1. Chronic Unpredictable Mild Stress (CUMS)

A chronic unpredictable mild stress (CUMS) model was used to evaluate the antidepressant effects of EOCP. Mild stressors were applied once daily between 8:00 and 10:00 a.m., except for those lasting 24 h, and were randomly distributed over 21 days. Stressors included forced swimming at 37 °C (5 min), wet bedding (24 h; 100 mL water + 50 g sand), food deprivation (24 h), tail pinching (15 min), water deprivation (24 h), forced swimming at 4 °C (5 min, followed by towel drying), and cage tilting at 45° (24 h). Depressive-like behavior was assessed using the forced swim test after the CUMS protocol [67,68].

#### 3.10.2. Forced Swim Test (FST)

The forced swim test (FST) is a widely used model to assess antidepressant-like activity in rodents [69,70]. A total of 60 mice were divided into six groups, with 10 animals per group. One hour after intraperitoneal administration of the treatments: vehicle 10 mL/kg (negative control), CUMS-induced mice without treatment (positive control), fluoxetine 20 mg/kg (standard drug) [28], and EOCP at doses of 50, 100, or 200 mg/kg. The mice were individually placed in a plastic cylinder (30 cm in height × 12 cm in diameter) filled with 20 cm of water at 23 ± 1 °C, for 6 min. The first 2 min were considered an acclimation phase, and behavior was recorded during the remaining 4 min [41,71]. Immobility was defined as the absence of active struggling, with the animal floating passively while making only minimal movements to keep its head above the water surface [72].

### 3.11. ADMET In Silico Prediction

The physicochemical properties of 15 molecules were initially predicted based on Lipinski’s five rules [73]. Then, the absorption, distribution, metabolism, excretion, and toxicity (ADMET) pharmacokinetic properties of each chemical compound were examined using SwissADME (http://www.swissadme.ch, accessed on 17 November 2025) and pkCSM (http://biosig.unimelb.edu.au/pkcsm, accessed on 17 November 2025) [74,75]. Then, potential CNS agents were selected using the boiled egg model, a predictive model of blood–brain barrier (BBB) permeability [76].

### 3.12. Molecular Docking Simulation

To discover the nature of intermolecular interactions and their binding energies, we applied molecular docking technology to candidate molecules and target proteins using Autodock Vina v1.2.1 [77]. The crystal structures of superoxide dismutase (ID: 2CDU), potassium channel (ID: 4UUJ), and presynaptic serotonin transporter (ID 5I6X) were obtained from the protein database (https://www.rcsb.org/) [78]. The proteins were then prepared using UCSF Chimera v1.18 by removing ligands, ions, and water molecules, and energy minimization was performed using Swiss-PdbViewer v4.1 [79]. Subsequently, they were parameterized by adding polar hydrogens, removing nonpolar hydrogens, and adding Gasteiger charges. All 3D structures of the ligands were downloaded from the PubChem database (https://pubchem.ncbi.nlm.nih.gov/) [80]. Ascorbic acid, diazepam, and imipramine were used as controls for antioxidant, anxiolytic, and antidepressant activity. The energy of the ligands was minimized using the MMFF94 force field in Avogadro software v1.2. Molecular docking was performed between the prepared proteins and the selected compounds that met the ADMET criteria. Grid boxes were defined for each target: a 25 Å × 25 Å × 25 Å grid centered at X = 33.91, Y = −21.69, and Z = −11.23 for the potassium channel from *Streptomyces lividans* (ID: 4UUJ); a 25 Å × 25 Å × 25 Å grid centered at X = −38.73, Y = −16.86, and Z = −1.75 for the presynaptic serotonin transporter (ID: 5I6X); and a 30 Å × 30 Å × 30 Å grid centered at X = 2.44, Y = 2.14, and Z = −1.08 for NADPH oxidase from *Lactobacillus sanfranciscensis* (ID: 2CDU). The docking protocol was validated by redocking the native ligand into the active site of each receptor [81,82]. The best docking poses were selected based on the lowest binding energy values, and the top-ranked conformations were visualized using UCSF Chimera v1.18 [83]. Two- and three-dimensional interaction profiles were analyzed using Discovery Studio Visualizer v2025 [84].

### 3.13. Statistical Analysis

All experiments were reported as means ± SD of three replicates (*n* = 3). Differences between the means were established using ANOVA-One way accompanied by Tukey’s range test. Normality of data distribution was visually inspected using GraphPad Prism version 8.0 (Intuitive Software for Science, CA, USA). The differences were considered statistically significant at *p* < 0.05.

## 4. Conclusions

This study provides neuropharmacological evidence supporting the traditional use of *Clinopodium pulchellum* (Panizara) in the management of nervous disorders. The essential oil exhibited anxiolytic- and antidepressant-like effects in validated in vivo models, with a significant behavioral response observed at the highest tested dose (200 mg/kg). However, the magnitude of the effect remained lower than that of standard reference drugs, indicating a moderate pharmacological activity. The phytochemical profile revealed a composition dominated by terpenoids such as β-caryophyllene and linalool, which are consistent with the chemotaxonomic characteristics of the Lamiaceae family and are likely contributors to the observed biological effects. In contrast, the presence of certain uncommon constituents should be interpreted cautiously and considered tentative, requiring further confirmation through complementary analytical approaches. In silico analysis, it supported the potential interaction of major terpenoids with targets related to neurotransmission, suggesting possible mechanisms underlying the observed effects. Overall, these findings contribute to the scientific validation of *C. pulchellum* as a source of bioactive compounds, although further pharmacological, analytical, and clinical studies are necessary to confirm its therapeutic potential.

## Figures and Tables

**Table 1:** Physicochemical properties and organoleptic characteristics of EOCP.

Organoleptic Characteristics	Description
Appearance	Clear liquid
Color	Light yellow
Smell	Aromatic, herbal
Taste	Spicy, cooling, slightly bitter
Physicochemical properties (at 20 °C)	Values
Relative Density	0.964 ± 0.02
Refractive index	1.487 ± 0.001
Acidity (pH)	5.4

**Table 2:** Chemical composition of the volatile compounds obtained from EOCP.

N.	Peak Name ^1,2^	RI ^3^	RI Lit ^4^	Relative Area (%)
1	ß-Pinene	980	978	1.22
2	ß-Mircene	992	991	0.82
3	D-Limonene	1024	1030	2.14
4	Eucalyptol	1032	1033	0.43
5	Trans-ß-Ocimene	1036	1047	0.26
6	ß-Ocimene	1050	1052	2.61
7	γ-Terpinene	1067	1062	0.35
8	3-Methyl-2-(2-methyl-2-butenyl) furan	1104	1104	42.04
9	Linalool	1095	1100	19.13
10	Caryophyllene	1419	1418	22.92
11	Humulene	1454	1450	2.55
12	Aromandendrene	1439	1440	0.33
13	Germacrene D	1481	1480	2.35
14	Alloaromandendrene	1460	1461	2.17
15	α-farnesene	1508	1503	0.69
Hydrocarbon monoterpenes				7.4%
Oxygenated monoterpenes				19.5%
Sesquiterpenes				31.0%

^1^ In order of elution on HP-5Ms. ^2^ Compounds identified according to RI and MS. ^3^ Retention indices established from the alkane series on the HP-5 MS capillary column (C9–C31). ^4^ Retention indices (RI Lit) were compared with literature values reported by Adams [14] and supported by mass spectral matching with NIST (v4.1) and Wiley (7th edition) libraries. Compounds were tentatively identified based on the combined use of RI and MS data.

**Table 3:** Anxiolytic-like effects of EOCP: time spent and number of entries in open and closed arms in the elevated plus maze (EPM) test.

Sample	Time in Seconds Mean ^1^ ± SEM		Number of Entries Mean ^1^ ± SEM	
	TOA	TCA	EOA	ECA
Vehicle	62.3 ^a^ ± 3.61	182.8 ^a^ ± 12.47	4.8 ^a^ ± 0.39	9.3 ^a^ ± 0.37
PTZ (20 mg/kg)	44.1 ^b^ ± 5.73	243.7 ^b^ ± 16.24	2.9 ^b^ ± 0.23	12.6 ^b^ ± 0.35
DZP (1 mg/kg)	207.2 ^c^ ± 12.57	75.2 ^c^ ± 5.16	12.8 ^c^ ± 0.79	7.3 ^c^ ± 0.58
EOCP (50 mg/kg)	93.3 ^d^ ± 4.21	167.8 ^d^ ± 7.78	4.6 ^a,d^ ± 0.34	9.9 ^d^ ± 0.48
EOCP (100 mg/kg)	121.5 ^a^ ± 4.31	152.3 ^e^ ± 5.36	6.5 ^e^ ± 0.31	8.4 ^a,c^ ± 0.30
EOCP (200 mg/kg)	170.7 ^f^ ± 4.93	135.4 ^f^ ± 4.66	9.1 ^f^ ± 0.50	7.8 ^e,c^ ± 0.46

^1^ Different superscripts in the same column showed significant differences (*p* < 0.05); PTZ = pentylenetetrazole; DZP = diazepam; EOCP = essential oil of *Clinopodium pulchellum;* TOA = time in open arms; TCA = time in closed arms; EOA = entries in open arms; ECA = entries in closed arms.

**Table 4:** Evaluation of antidepressant-like effects of EOCP based on immobility time in the forced swim test (FST) after exposure to chronic unpredictable mild stress (CUMS) in mice.

Sample	Immobility Time (s) Mean ^1^ ± SEM
Vehicle (10 mL/kg)	170.9 ^a^ ± 5.47
CUMS (21 days)	204.3 ^b^ ± 8.26
FXT (20 mg/kg)	86.5 ^c^ ± 2.91
EOCP (50 mg/kg)	167.9 ^a^ ± 5.32
EOCP (100 mg/kg)	155.1 ^a^ ± 5.20
EOCP (200 mg/kg)	128.7 ^d^ ± 4.41

^1^ Different superscripts in the same column showed significant differences (*p* < 0.05); CUMS = exposure to chronic unpredictable mild stress; FXT = fluoxetine; EOCP = essential oil of *Clinopodium pulchellum*.

**Table 5:** Prediction of physico-chemical parameters of 15 chemical compounds of EOCP.

N.	Compounds	MW ≤ 500 Da	HBD ≤ 5	HBA ≤ 10	cLog P_o/w_ ≤ 5	MR 40–130	N° Violations ≤ 2	Drug-Likeness
1	3-Methyl-2-(2-methyl-2-butenyl) furan	Yes	Yes	Yes	Yes	Yes	0	Yes
2	α-farnesene	Yes	Yes	Yes	Yes	Yes	0	Yes
3	Alloaromandendrene	Yes	Yes	Yes	Yes	Yes	0	Yes
4	Aromandedrene	Yes	Yes	Yes	Yes	Yes	0	Yes
5	β-myrcene	Yes	Yes	Yes	Yes	Yes	0	Yes
6	β-ocimene	Yes	Yes	Yes	Yes	Yes	0	Yes
7	β-pinene	Yes	Yes	Yes	Yes	Yes	0	Yes
8	Caryophyllene	Yes	Yes	Yes	Yes	Yes	0	Yes
9	D-limonene	Yes	Yes	Yes	Yes	Yes	0	Yes
10	Eucalyptol	Yes	Yes	Yes	Yes	Yes	0	Yes
11	Germacrene D	Yes	Yes	Yes	Yes	Yes	0	Yes
12	Humulene	Yes	Yes	Yes	Yes	Yes	0	Yes
13	Linalool	Yes	Yes	Yes	Yes	Yes	0	Yes
14	Trans-β-ocimene	Yes	Yes	Yes	Yes	Yes	0	Yes
15	γ-terpinene	Yes	Yes	Yes	Yes	Yes	0	Yes

**Table 6:** Prediction of pharmacokinetic properties of ADMET for 15 chemical compounds of EOCP.

N.	Compounds	Absorption	Distribution		Metabolism							Excretion	Toxicity		
		Intestinal Absorption (Human)			Substrate Inhibitor							Clearance Total)	AMES Toxicity	Hepatotoxicidad	Skin Sensitization
					Cytochromes										
			BBB Permeability	CNS Permeability	2D6	3A4	1A2	2C19	2C9	2D6	3A4				
		(% Absorbido)	(Log BB)	(Log PS)	Categorical (Yes/No)							(Log mL/min/kg)	Categorical (Yes/No)		
1	3-Methyl-2-(2-methyl-2-butenyl) furan	97.926	0.551	−1.895	No	No	Yes	No	No	No	No	0.449	No	No	Yes
2	α-farnesene	96.065	0.746	−1.168	No	No	Yes	No	No	No	No	1.608	No	No	Yes
3	Alloaromandendrene	95.302	0.822	−1.769	No	Yes	No	No	No	No	No	0.926	No	No	No
4	Aromandedrene	95.302	0.822	−1.769	No	Yes	No	No	No	No	No	0.926	No	No	No
5	β-myrcene	94.696	0.781	−1.902	No	No	No	No	No	No	No	0.438	No	No	No
6	β-ocimene	94.726	0.761	−1.848	No	No	No	No	No	No	No	0.441	No	No	No
7	β-pinene	95.525	0.818	−1.857	No	No	No	No	No	No	No	0.030	No	No	No
8	Caryophyllene	94.845	0.733	−2.172	No	No	No	No	No	No	No	1.088	No	No	Yes
9	D-limonene	95.898	0.732	−2.370	No	No	No	No	No	No	No	0.213	No	No	Yes
10	Eucalyptol	96.505	0.368	−2.972	No	No	No	No	No	No	No	1.009	No	No	Yes
11	Germacrene D	95.590	0.723	−2.138	No	No	No	No	No	No	No	1.420	No	No	Yes
12	Humulene	96.611	0.492	−2.181	No	No	Yes	No	No	No	No	1.080	No	No	Yes
13	Linalool	95.910	0.502	−1.968	No	No	Yes	No	No	No	No	0.456	No	No	Yes
14	Trans-β-ocimene	97.624	0.642	−1.474	No	No	Yes	No	No	No	No	0.452	No	No	No
15	γ-terpinene	98.048	0.565	−1.659	No	No	Yes	No	No	No	No	0.227	No	No	No

**Table 7:** Binding affinity scores of fifteen selected molecules and standard drugs in molecular docking interactions.

N.	ID PubChem	Compound	Anxiolytic PDB: 4UUJ (kcal/mol)	Antidepressant PDB: 5I6X (kcal/mol)	Antioxidant PDB: 2CDU (kcal/mol)
1	84825	3-Methyl-2-(2-methyl-2-butenyl) furan	−4.3	−6.3	−5.3
2	5281516	α-farnesene	−4.0	−7.4	−6.1
3	10899740	Alloaromandendrene	−5.1	−6.3	−6.8
4	91354	Aromandedrene	−5.5	−7.9	−6.8
5	31253	β-myrcene	−4.1	−5.9	−5.3
6	5281553	β-ocimene	−4.2	−6.1	−5.3
7	440967	β-pinene	−4.3	−5.9	−5.1
8	6429274	Caryophyllene	−5.3	−7.8	−6.7
9	439250	D-limonene	−4.7	−6.4	−5.4
10	2758	Eucalyptol	−4.4	−5.8	−5.3
11	24771782	Germacrene D	−5.0	−7.8	−6.4
12	5281520	Humulene	−5.2	−8.1	−6.2
13	67179	Linalool	−3.2	−3.5	3.4
14	5281553	Trans-β-ocimene	−4.2	−6.1	−5.3
15	7461	γ-terpinene	−4.8	−6.4	−5.7
16	3016	Diazepam	−6.0	-	
17	3696	Imipramine	-	−8.7	-
18	54670067	Acid ascorbic	-	-	−6.4

## Data Availability

All data generated or analyzed during this study are included in this published article.

## Abbreviations

The following abbreviations are used in this manuscript:

EOCP	essential oil of *Clinopodium pulchellum*
DPPH	2,2-diphenyl-1-picrylhydrazyl
ABTS	2,2′-azino-bis-3-ethylbenzothiazoline-6-sulfonic acid
EPM	elevated plus maze
CUMS	chronic unpredictable mild stress
FST	forced swim test
ADMET	absorption, distribution, metabolism, excretion, and toxicity
